# US Population Data for 94 Identity-Informative SNP Loci

**DOI:** 10.3390/genes14051071

**Published:** 2023-05-12

**Authors:** Kevin M. Kiesler, Lisa A. Borsuk, Carolyn R. Steffen, Peter M. Vallone, Katherine B. Gettings

**Affiliations:** National Institute of Standards and Technology, 100 Bureau Drive, Mailstop 8314, Gaithersburg, MD 20899, USA

**Keywords:** single nucleotide polymorphism, human identification, microhaplotype, next generation sequencing

## Abstract

The US National Institute of Standards and Technology (NIST) analyzed a set of 1036 samples representing four major US population groups (African American, Asian American, Caucasian, and Hispanic) with 94 single nucleotide polymorphisms (SNPs) used for individual identification (iiSNPs). The compact size of iiSNP amplicons compared to short tandem repeat (STR) markers increases the likelihood of successful amplification with degraded DNA samples. Allele frequencies and relevant forensic statistics were calculated for each population group as well as the aggregate population sample. Examination of sequence data in the regions flanking the targeted SNPs identified additional variants, which can be combined with the target SNPs to form microhaplotypes (multiple phased SNPs within a short-read sequence). Comparison of iiSNP performance with and without flanking SNP variation identified four amplicons containing microhaplotypes with observed heterozygosity increases of greater than 15% over the targeted SNP alone. For this set of 1036 samples, comparison of average match probabilities from iiSNPs with the 20 CODIS core STR markers yielded an estimate of 1.7 × 10^−38^ for iiSNPs (assuming independence between all 94 SNPs), which was four orders of magnitude lower (more discriminating) than STRs where internal sequence variation was considered, and 10 orders of magnitude lower than STRs using established capillary electrophoresis length-based genotypes.

## 1. Introduction

When highly compromised biological samples are encountered, DNA template molecules may be too short for efficient amplification of any region beyond 100 to 200 bases in length using standard polymerase chain reaction (PCR) amplification. The largest PCR amplicons are most adversely affected by fragmented or inhibited samples, leading to a characteristic downward trend in detection intensity with increasing amplicon size. For the most highly compromised DNA samples, few or no STR markers will amplify in a typical human identification (HID) multiplex. In such cases, markers with smaller PCR amplicons are better suited to HID applications [1,2,3,4]. Single nucleotide polymorphisms (SNPs) can be assayed with short (<150 bp) PCR fragment lengths, increasing the likelihood of successful amplification of sufficient loci for comparison with reference samples. Modern DNA sequencing methods make analysis of large SNP panels an attractive method for HID testing challenging samples such as highly degraded DNA or complex kinship cases [5,6,7,8,9,10,11,12,13].

Prior to the introduction of next-generation sequencing methods at the turn of the millennium, obtaining DNA sequences was laborious. For forensic applications, DNA sequences could be analyzed individually using Sanger sequencing [14], or small multiplexes of target SNPs could be assayed using single-base extension [5]. These methods relied on relatively low-throughput techniques such as slab gels or capillary electrophoresis for detection. However, in 2010, Ion Torrent Systems, Inc. (San Francisco, CA, USA) introduced the Ion Personal Genome Machine (PGM) [15], and in 2011, Illumina Inc. (San Diego, CA, USA) marketed the MiSeq instrument [16]. Both benchtop systems offered scale and cost amenable to forensic applications. Importantly, the increased scale of sequencing parallelized reactions and detection enabled the generation of large quantities of DNA sequence information. To complement these sequencing platforms, commercial vendors have developed and validated assays with both traditional (STR) and newer (SNP) marker types for HID purposes [11]. A key aspect of introducing new markers in the legal framework for HID is access to appropriate population genetic data to formulate weight-of-evidence estimates given the proposition of a one-to-one match with an investigated sample and an individual [17,18,19,20,21,22,23,24,25]. The ForenSeq DNA Signature Prep Kit (Verogen, San Diego, CA, USA) is a large multiplex PCR assay designed for use with the MiSeq FGx (Verogen) to produce DNA sequences from both STR and SNP markers. The focus of this paper is the 94 identity-informative SNP markers (iiSNPs) present in the ForenSeq DNA Signature Prep Kit (both DNA Primer Mix A (DPMA) and B (DPMB)). The 94 iiSNP markers in the ForenSeq DNA Signature Prep Kit were originally proposed as separate panels in the literature [5,26,27] for identity informativeness because they occur in relatively balanced allelic proportions in many worldwide populations.

We present the sequencing results and allele frequencies for these 94 iiSNPs, and additional variation contained in amplified regions flanking the targeted SNPs, in four sets of samples representative of the US population (African American, Asian American, Caucasian, and Hispanic). This work facilitates statistical calculations in investigative DNA identification efforts where the populations characterized here are relevant.

## 2. Materials and Methods

### 2.1. Samples and Sequencing

Anonymous liquid blood samples with self-reported ancestries were purchased from Interstate Blood Bank (Memphis, TN, USA) and Millennium Biotech, Inc. (Ft. Lauderdale, FL, USA) or provided by DNA Diagnostics Center (Fairfield, OH, USA) as buccal swabs from paternity testing samples anonymous to NIST. A total of 1036 samples were included in this study, the same samples previously reported by our group [28,29,30], divided among four US populations (population names as designated during sample collection): African American (N = 342), Asian American (N = 97), Caucasian (N = 361), and Hispanic (N = 236). Throughout this paper, N = 1036 is used to reference the number of samples, whereas 2072 is the implied number of chromosomes. All work presented has been reviewed and approved by the NIST Research Protections Office under protocol # MML-16-0080.

Samples were analyzed as previously described [29] with the ForenSeq DNA Signature Prep Kit (Verogen, San Diego, CA, USA) used for library construction and sequencing on a MiSeq FGx instrument (Verogen). Modifications were made to the manufacturer’s recommended procedure to improve profile recovery (e.g., increasing DNA input, see [29] for an extensive description of methods).

### 2.2. Data Analysis and Interpretation

#### 2.2.1. Universal Analysis Software (UAS)

Primary data analysis was performed using Universal Analysis Software (UAS) version 1.3 (Verogen) [11]. SNP genotypes and sequencing coverage data were exported from the UAS for downstream evaluation in Excel (Microsoft, Redmond, WA, USA). Analytical and stochastic thresholds were applied in evaluating genotype calls as follows: greater than 30 reads were required for homozygous genotypes, whereas for heterozygous calls greater than 10 reads for each of two alleles were required. Allele coverage ratio (ACR), defined here as the number of sequencing reads from the allele with lower coverage divided by that of the allele with higher coverage, was required to be greater than 20% for heterozygous calls.

#### 2.2.2. In-House Analysis

As an alternate approach to the UAS, an in-house method incorporating STRait Razor v3.0 (SR3) [31] was used to analyze the fastq files, and the resulting target SNP genotypes were compared with UAS genotypes. Regions of DNA concomitantly sequenced with the target SNP (“flanking regions”) were also evaluated for additional polymorphisms. STRait Razor relies on a configuration (“config”) file to define the analyzed sequence regions. The config file used in this analysis was the default ForenSeq config file provided with SR3, ForenSeqv1.27.config (https://github.com/Ahhgust/STRaitRazor, accessed on 26 August 2022). Allele calling thresholds used with UAS data (above) were also applied to the results of this in-house analysis.

### 2.3. Calculation of Allele Frequencies and Forensic Statistics

The finalized SNP call set was used as input for STRAF 2.0 [32] for calculation of allele frequencies and forensic statistics: power of discrimination (PD), gene diversity (GD), probability of matching (PM), polymorphism information content (PIC), power of exclusion (PE), and typical paternity index (TPI).

### 2.4. Testing for Linkage Disequilibrium and Hardy–Weinberg Equilibrium

Arlequin 3.5 [33] was used for testing linkage disequilibrium with 10,000 permutations and two initial conditions for Expectation Maximization. Testing for Hardy–Weinberg equilibrium was performed in Arlequin with 1 × 10^6^ Markov chain steps and 1 × 10^5^ dememorization steps on a locus-by-locus basis.

### 2.5. Calculation of Effective Alleles (A_e_)

The number of effective alleles for each locus was calculated using the equation: A_e_ = 1/(p^2^ + q^2^), where p and q are the frequencies of the two allelic states for a biallelic SNP. For each locus, A_e_ was calculated for each population tested using population-specific allele frequencies from the dataset.

### 2.6. Calculation of Random-Match Probabilities (RMP)

Random-Match Probabilities for individual SNP profiles were calculated using frequency data from the 1000 Genomes Project [34] for the 94 iiSNPs in the ForenSeq DNA Signature Prep Kit, assuming independence between all markers. Similarly, RMPs were calculated for STR loci using either CE-based frequencies [28] or sequence-based frequencies [29]. For purposes of comparison, only the 20 CODIS Core STR loci were included in the STR-based RMP calculations. Individual RMP, average RMP, and standard deviation for the full 1036 sample set were calculated in Excel.

## 3. Results and Discussion

### 3.1. Call Rate, Sequencing Coverage, and Heterozygote Balance

A total of 97,384 genotype calls can be made for a population of 1036 samples at 94 loci. In the current study, 96,889 genotypes are reported after manual curation of the UAS genotype calls and comparison with an orthogonal analysis method (Section 3.4) for an overall genotype call rate of 99.5%. The remaining 495 genotypes failed to meet the minimum criteria described above, primarily due to insufficient sequencing coverage. Two loci, rs1031825 and rs1736442, had uncharacteristically high no-call rates (i.e., genotype dropout) of 19.1% and 18.6%, respectively, accounting for 391 of the 495 UAS dropouts. These two amplicons had the lowest average sequencing coverage of the 94 iiSNPs in this data set (Figure S1). Recent literature also notes these loci as low performing [25]. The remaining no-calls were distributed among 19 SNP loci, each with dropout rates of less than 1% of the 1036 genotypes (except for rs7041158, which had a dropout rate of 1.4%). Median sequencing coverage per locus was 553× and ranged from nearly 5000× to 50×, spanning two orders of magnitude (Figure S1). Care should be taken in interpreting sequencing coverage outcome here because modification of the manufacturer’s recommended procedure, in the form of increased DNA input for low-performing samples, may have led to variability from sample to sample.

ACR was generally consistent across loci (Figure S1), with a mean of 0.86 ± 0.07 (range 0.43 to 0.92). Two loci had mean ACR values below 0.6 (rs6955448 and rs338882). These loci had the most skewed ACR distributions and were observed to have similarly low ACR values in recent literature [21,25,35,36]. Locus rs6955448 exhibited lower average coverage for the alternate allele [T] (245.33× ± 123.54×) versus the reference allele [C] (573.68× ± 289.43×), resulting in an average ACR of 0.43 ± 0.09 in this study. Recent work by Davenport et al. [25] identified sequence variation in the primer binding region of locus rs6955448 as a likely cause of imbalanced sequencing coverage.

Locus rs338882 exhibited lower average coverage for the reference allele [C] (73.45× ± 41.82×) versus the alternate allele [T] (146.82× ± 81.47×), with an average ACR of 0.51 ± 0.13 (please note: the UAS version 1.3 reports this SNP locus on the reverse strand of the GRCh38 genome [C/T]; here we use the genotype given by UAS on the reverse strand to aid current UAS operators, whereas the GRCh38 forward strand genotype is [G/A]; Table S1 includes the UAS v1.3 strand orientation of each iiSNP). Investigation into imbalanced sequence coverage at locus rs338882 by King et al. [21] was unable to conclusively determine a causal factor.

### 3.2. Allele Frequencies, Forensic Statistics, Linkage Disequilibrium, and Hardy–Weinberg Calculations

Allele frequencies, forensic statistics, and linkage disequilibrium calculations were performed overall and by population for each of the 94 iiSNPs (Table S1). Reported allelic frequency values for 94 iiSNPs are consistent with the strand orientation output by the UAS version 1.3 (as noted in Table S1). The maximum sample size for frequency calculations is 2072 alleles for the full 1036 sample set; some loci had fewer reported alleles (Table S1) when quality metrics did not meet minimum allele calling thresholds.

Forensic statistics (Gene Diversity (GD), Polymorphism Information Content (PIC), Probability of Matching (PM), Power of Discrimination (PD), Observed Heterozygosity (Hobs), Power of Exclusion (PE), and Typical Paternity Index (TPI)) calculated in STRAF [32] are presented in Table S2. Forensic parameters are displayed graphically as boxplots for each population in Figure S2, allowing the reader to explore trends in the data where we observe some dispersion in values for a small number of loci. Upon closer examination of dispersed data points, no clear patterns emerged in terms of any one marker performing differently across all populations or all categories of parameters.

Testing for Linkage Disequilibrium in Arlequin returned 913 SNP pairs with exact T-test values below Arlequin’s default significance level (*p* < 0.05). When performing numerous pairwise comparisons, it is expected that some level of type-1 errors (i.e., false positives) will be observed due to random chance when using a significance threshold suitable for discovering linkage disequilibrium indicators. This is exemplified by the observation that, of the 913 SNP pairs with *t*-test *p*-values < 0.05, only 36 pairs are physically located on the same chromosome (Table S3). After applying the conservative Bonferroni correction, only one syntenic pair remains statistically significant, rs1355366 and rs1357617 (*p* < 0.0001 only in the Caucasian category); these loci are physically separated by 190 Mb of DNA sequence on Chromosome 3.

Hardy–Weinberg equilibrium testing in Arlequin produced a small number of loci (between one and four, depending on population) with *p*-values < 0.05 (Table S4). Again, after applying the Bonferroni correction method, no *p*-values remained statistically significant.

### 3.3. Effective Alleles per Locus (A_e_)

**Figure 1:**
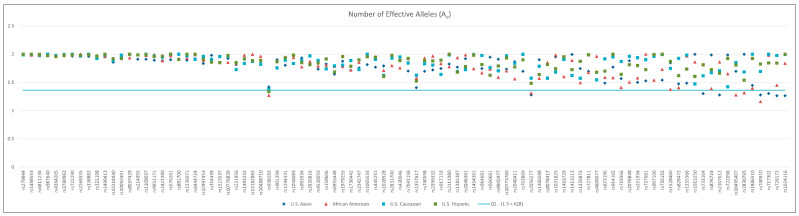
Number of effective alleles for 94 iiSNPs in four US populations. Each point on the plot represents the A_e_ value from one of four populations in the study. A reference line is drawn at the first quartile minus 1.5 times the interquartile range of A_e_ values.

The number of effective alleles per locus is shown in Figure 1, with each data point representing the value within a population. Loci rs938283, rs1357617, and rs2056277 produced lower A_e_ values for all populations. The original literature from the SNPforID Consortium recommending these three loci for identity-informative purposes [5] characterized performance in population samples collected in Denmark, Greenland, Somalia, Turkey, China, Germany, Taiwan, Thailand, and Japan, where allele frequencies may differ from those studied here. Fluctuations in A_e_ values reflect slight allele frequency variation among populations from different regions of the globe. This highlights the importance of using appropriate population frequency data for weight-of-evidence calculations.

### 3.4. Concordance with an Orthogonal Analysis Pipeline

Comparison of UAS genotype calls with those generated by an in-house analysis method incorporating SR3 yielded 97,222 concordant genotype calls from 97,384 possible genotypes (99.8% concordant). Genotypes that did not satisfy minimum thresholds by both analysis pipelines were considered concordant with the UAS calls (thus, the number of concordant genotypes is higher than the number of UAS genotype calls). The remaining 162 discordant genotypes were mostly no-calls by the SR3, where the presence of errors at various positions within sequencing reads excluded those reads from the count of reads corresponding to the ‘correct’ genotype call. This mechanism caused the read count to drop below allele calling thresholds in 154 cases. In eight instances, the SR3 analysis recovered genotypes that did not satisfy minimum thresholds in the UAS call set.

**Figure 2:**
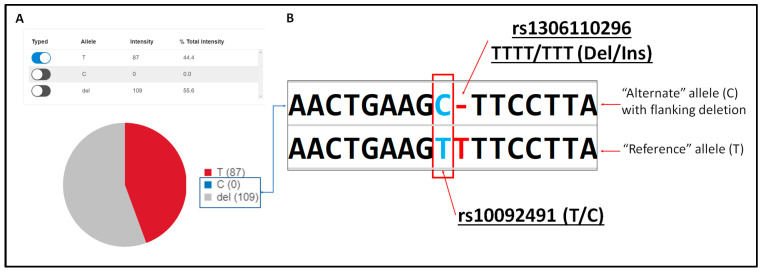
(**A**) Genotype of homozygous T called at rs10092491 produced by the UAS; 109 reads were interpreted as deletions while no reads were interpreted as C base calls, (**B**) sequence alignment showing a heterozygous C allele with deletion of one T base (rs1306110296) from the adjacent homopolymer stretch.

Close inspection of the genotypes from the two analyses revealed an incorrect UAS genotype call for one sample at SNP locus rs10092491. This sample was typed by the UAS as homozygous T, whereas the sequence analyzed by the in-house analysis resulted in a heterozygous T/C genotype. The UAS interpreted 109 of 196 reads (>50%) as deletions (Figure 2), which did not trigger any automatic flag to draw the attention of the reviewer. This target SNP (rs10092491 T > C) is adjacent to a homopolymer of up to four T bases containing a known low-frequency deletion (rs1306110296, frequency < 0.0001 in GnomAD [37]). The UAS produced a deletion call when the C allele of rs10092491 was present in combination with a deleted T from the homopolymer (Figure 2), presumably as an artifact of the alignment method used by the UAS. This genotype was adjusted in the final call set to reflect the correct heterozygote call (T/C) in this sample for downstream population genetic analysis.

### 3.5. Microhaplotypes

The region containing SNP rs10092491 with an adjacent deletion represents a microhaplotype (MH, defined in the context of this study as any flanking sequence not matching the GRCH 38 reference genome, in combination with the UAS target SNP) obtained with the in-house analysis method. Further examination of the output of genotype calls from the in-house analysis indicated MHs of one or more flanking SNPs (plus the UAS target SNP) in 74 of the 94 iiSNP amplicons in the ForenSeq DNA Signature Prep Kit (Table S5). Of these 74 loci with MHs, 36 loci had one or more MH alleles labeled ‘novel’ (Table S6) in the output from the underlying SR3 database (downloaded 26 August 2022), indicating these MHs had not been observed in the original population study from which the SR3 database was established [19]. In total, 155 unique MH alleles accounted for 5198 flanking sequence variant observations, or 5.3% of 97,222 of the concordant genotype calls.

**Figure 3:**
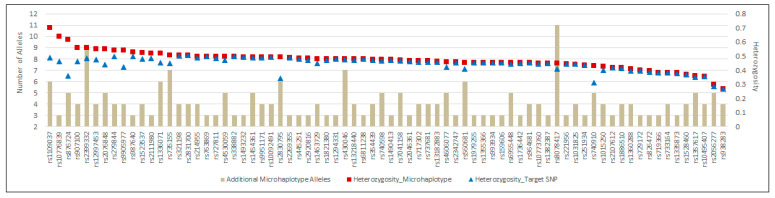
The additional number of alleles (left axis, bars) created by flanking sequence microhaplotypes and observed heterozygosity (right axis) when considering UAS target SNPs (triangles) versus microhaplotypes (squares) in 74 loci where flanking sequence variation was observed.

Of the 74 loci with microhaplotypes identified, four microhaplotypes associated with target SNP loci rs876724, rs1109037, rs10776839, and rs2830795 (Figure 3) exhibited the largest increases H_obs_ (15% to 26%) as compared to the target SNPs alone, consistent with the published literature [20,21]. Another eight microhaplotypes had increased H_obs_ from between 5% and 15%, while the remaining 62 loci with microhaplotypes exhibited low (<5%) or no increase in H_obs_. Some microhaplotypes were observed at low frequency; 36 loci had singleton observations (accounting for 64 MH alleles) representing private variation which had negligible impact on H_obs_ in this study.

### 3.6. Calculation of Random-Match Probabilities

**Figure 4:**
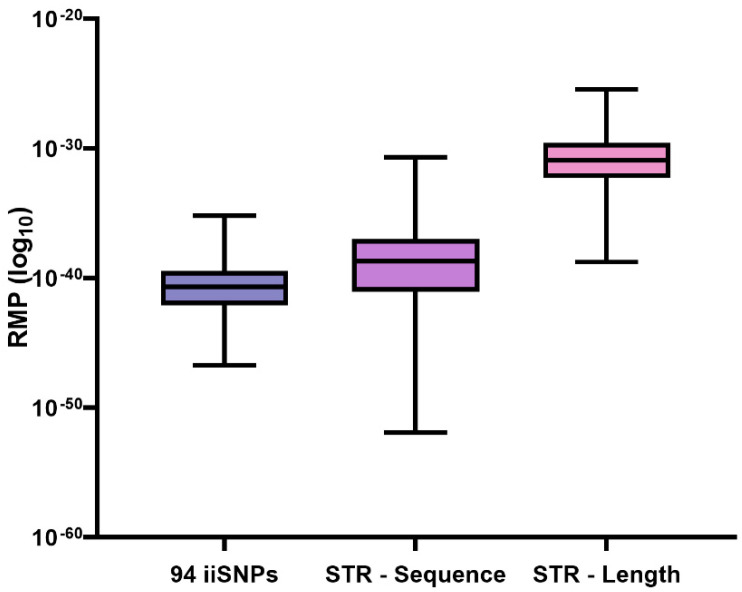
Boxplot of RMP values for the 1036 sample set with 94 iiSNPs, 20 CODIS sequence-based STRs, and 20 CODIS length-based STRs. The box represents the first and third quartiles with a line at the median value. Whiskers represent minimum and maximum values.

For the 1036 samples in the study (which were previously described for sequence-based STRs [29] and length-based STRs [28]), the RMP values calculated from the 94 iiSNPs (mean = 1.68 × 10^−38^) were approximately four orders of magnitude lower (more discriminating) than the RMP values calculated from the 20 CODIS core sequence-based STRs (mean = 2.36 × 10^−34^). Further, the 94 iiSNPs and sequence-based STRs outperformed length-based STR RMP calculations (mean = 1.02 × 10^−28^) by ten and six orders of magnitude, respectively (Figure 4).

## 4. Conclusions

An overall call rate of 99.5% was observed for these 94 iiSNPs in this study of 1036 samples. While some variation in sequencing coverage was observed, modifications of the recommended procedure were made in the interest of improved profile recovery from underperforming DNA templates. Coverage metrics in this study may not be representative of routine use of the ForenSeq DNA Signature Prep Kit. The two SNP loci with the lowest average coverage, rs1031825 and rs1736442, produced most of the genotype dropouts (19.1% and 18.6% dropout rates, respectively). The assay exhibited mostly uniform coverage for heterozygous genotype calls (ACR). Two loci had frequently imbalanced allele coverage: rs6955448 and rs338882.

Forensic parameters were mainly consistent across all iiSNP markers in the multiplex, with some slight deviations observed in rare instances for specific populations. One locus, rs938283, exhibited minor skew in the number of effective alleles (A_e_) in all populations in the study. Analysis of A_e_ values (Figure 1) across the 94 iiSNPs revealed some loci with allelic frequency variation among populations from different regions of the world.

Linkage disequilibrium analysis indicated signals of pairwise disequilibrium in 36 pairs of SNPs co-located on the same chromosome. Applying the Bonferroni correction eliminates all but one syntenic pair from statistical significance. This pair is physically separated by nearly the entirety of chromosome 3; therefore, it is unlikely this LD signal arises from a lack of recombination between these two loci. However, further exploration of the LD *p*-values reported here and comparison with results from other studies is encouraged prior to statistically combining these 94 iiSNPs, which arose from independently characterized panels.

Analysis with an alternate bioinformatic method brought to light a rare deletion (rs1306110296) in the short homopolymer adjacent to the target SNP locus rs10092491. This ostensibly caused misalignment by the UAS analysis algorithm, resulting in an incorrect homozygous genotype call. While this low-frequency deletion was observed in only one sample in this population study of 1036 individuals, the presence of a high number of reads interpreted as deletions could serve as an indicator of this or similar artifacts, which may not be automatically flagged by the UAS.

Flanking region variation comprising microhaplotypes within the iiSNP amplicons in the ForenSeq DNA Signature Prep Kit suggests the possibility of extracting more information from the kit’s SNP content by analysis of the full sequence string. Microhaplotypes associated with target SNP loci rs876724, rs1109037, rs10776839, and rs2830795, are likely to routinely provide additional information. Some flanking variation seen at low frequency may be of benefit to kinship analysis. The full complement of 94 biallelic iiSNPs produces extremely low random-match probabilities, making complex additional analyses beyond the UAS optional. The iiSNP random-match probabilities are comparable to values calculated for sequence-based alleles of STRs, both of which outperformed length-based STRs by several orders of magnitude in this study.

The NIST 1036 sample set was used here to characterize performance of the iiSNP content of the ForenSeq DNA Signature Prep Kit in terms of technical performance, population genetic parameters, and direct comparison with RMP values from STR markers by length and sequence. In the absence of corroborating genotype data for the 94 iiSNP loci, we used a parallel analysis method to improve the accuracy of genotype curation and to provide frequency information for sequence variation in flanking regions of the amplicons. The use of the NIST 1036 sample set in this study adds to the extensive forensic marker information available for these samples and maintains their relevance in facilitating technology transition.

## Data Availability

All SNP genotypes are available from the NIST Public Data Repository: US population data for human identification markers (doi:10.18434/t4/1500024).

## Supplementary Materials

The following supporting information can be downloaded at https://www.mdpi.com/article/10.3390/genes14051071/s1 and at the NIST Public Data Repository: US population data for human identification markers (doi:10.18434/t4/1500024); Figure S1: Sequencing metrics for 94 ForenSeq Signature Prep Kit identity-informative SNP loci in a population of 1036 samples; Figure S2: Forensic parameters (Genetic Diversity (GD), Polymorphism Information Content (PIC), Random-Match Probability (PM), Power of Discrimination (PD), Observed Heterozygosity (Hobs), Power of Exclusion (PE), and Typical Paternity Index (TPI)) shown in boxplot format for each of four populations studied ((A)African American, (B) Asian American, (C) Caucasian, and (D) Hispanic) [38,39]; Table S1: Allele Frequencies; Table S2: Forensic Parameters; Table S3: Linkage Disequilibrium; Table S4: Hardy–Weinberg Equilibrium; Table S5: Microhaplotype Frequencies; Table S6: Novel Microhaplotypes.
