# Supplementary material for: US Population Data for 94 Identity-Informative SNP Loci
Source: Genes (Basel). 2023 May 12;14(5):1071. doi: 10.3390/genes14051071 (PMC10217833; doi:10.3390/genes14051071)
Supplement: Supplementary file 1 [file genes-14-01071-s001.zip › Supplemental Figures and Captions.pdf]

Figure S1: Sequencing metrics for 94 ForenSeq Signature Prep Kit identity informative SNP loci in a population of 1036 samples: (A) average sequencing coverage for each locus, with mean value of 553 × (dashed red line), with a maximum of 4945.9 × ± 512.1 × and a minimum of 49.5 × ± 545.3 × (B) allele coverage ratio had a mean value of 0.86 ± 0.07, a cutoff of 0.6 was chosen (dashed blue line) for further investigation of loci with notably lower ACR.

A

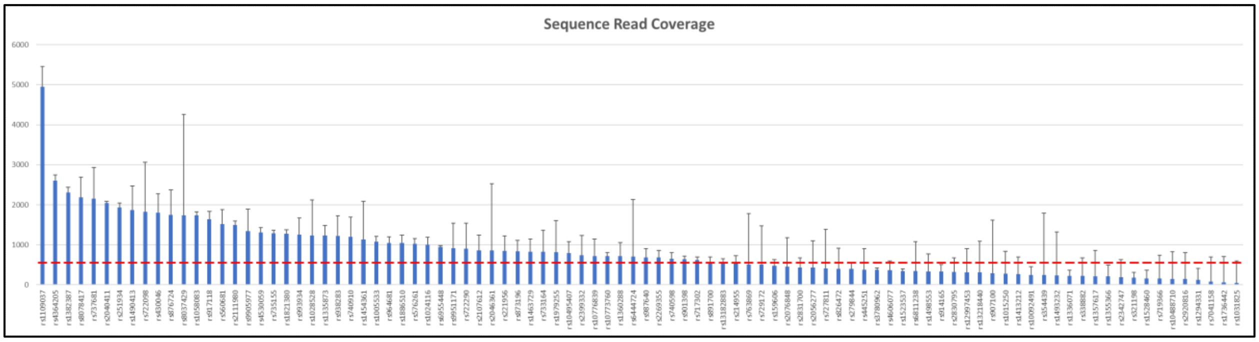

B

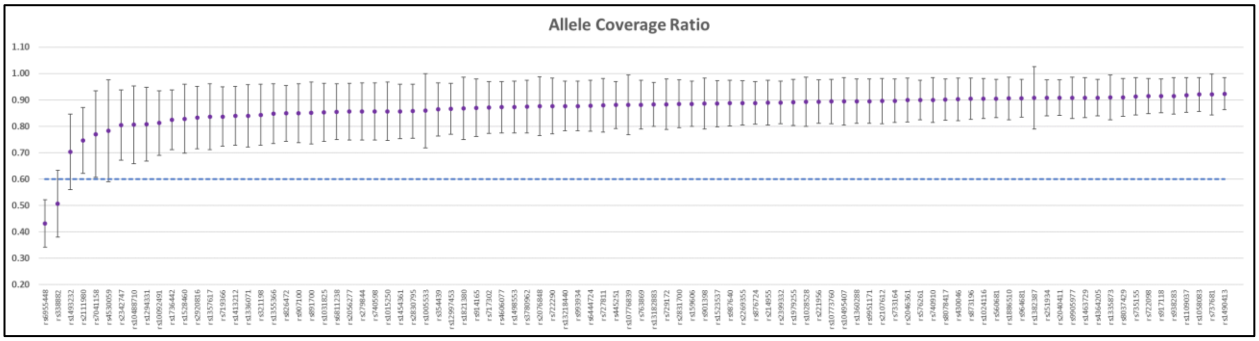

Figure S2: Forensic parameters (Genetic Diversity (GD), Polymorphism Information Content (PIC), Random Match Probability (PM), Power of Discrimination (PD), Observed Heterozygosity (Hobs), Power of Exclusion (PE), and Typical Paternity Index (TPI)) shown in boxplot format for each of four populations studied ((A)African American, (B) Asian American, (C) Caucasian, and (D) Hispanic). In each plot the box extends from the 25th (Q1) to the 75th (Q3) percentiles, with a line drawn at the mean value. The whiskers are drawn according to the method described by J.W. Tukey [1,2].

A

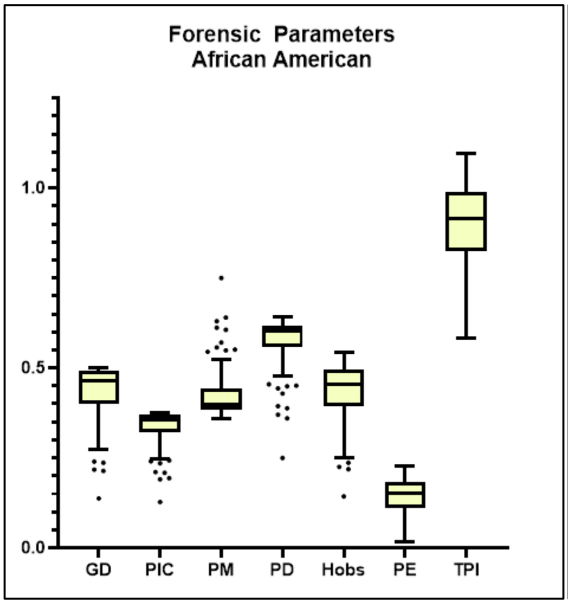

B

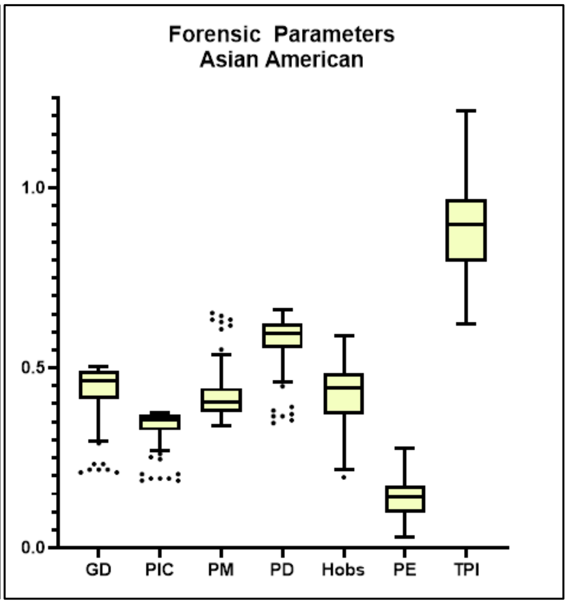

C

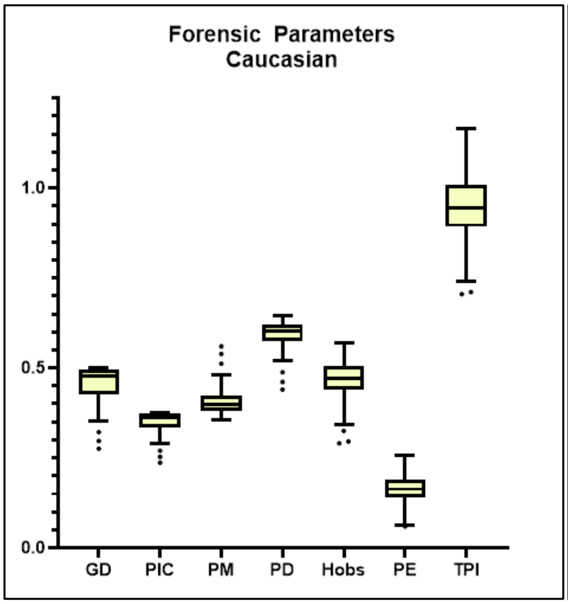

D

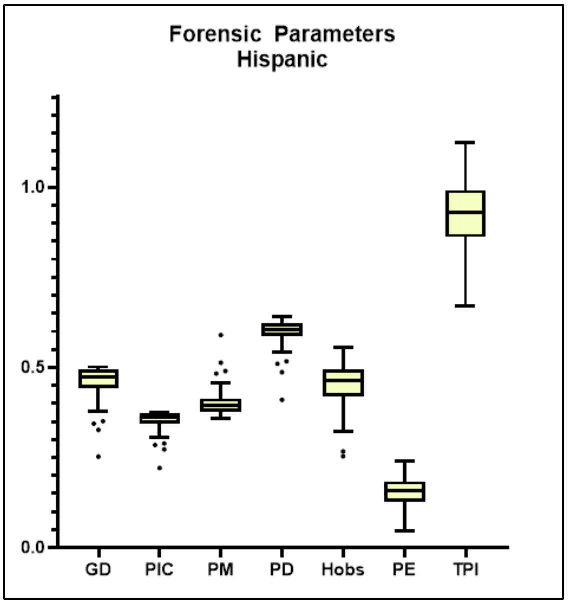

References:

1. Tukey, J.W. (1977). Exploratory data analysis. Reading, MA: Addison-Wesley.
2. McGill, R., Tukey, J. W. and Larsen, W. A. (1978) Variations of box plots. The American Statistician 32, 12-16.
