# Supplementary figures and images for: US Population Data for 94 Identity-Informative SNP Loci
Source: Genes (Basel). 2023 May 12;14(5):1071. doi: 10.3390/genes14051071 (PMC10217833; doi:10.3390/genes14051071)

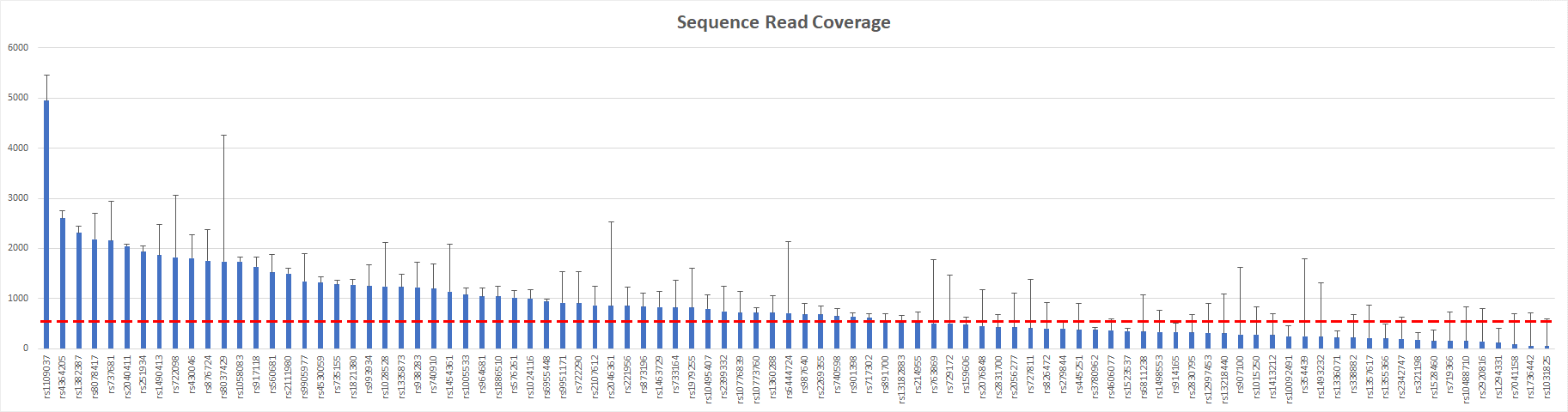

Supplement: Supplementary file 1 [file genes-14-01071-s001.zip › Figure S1 A.png]

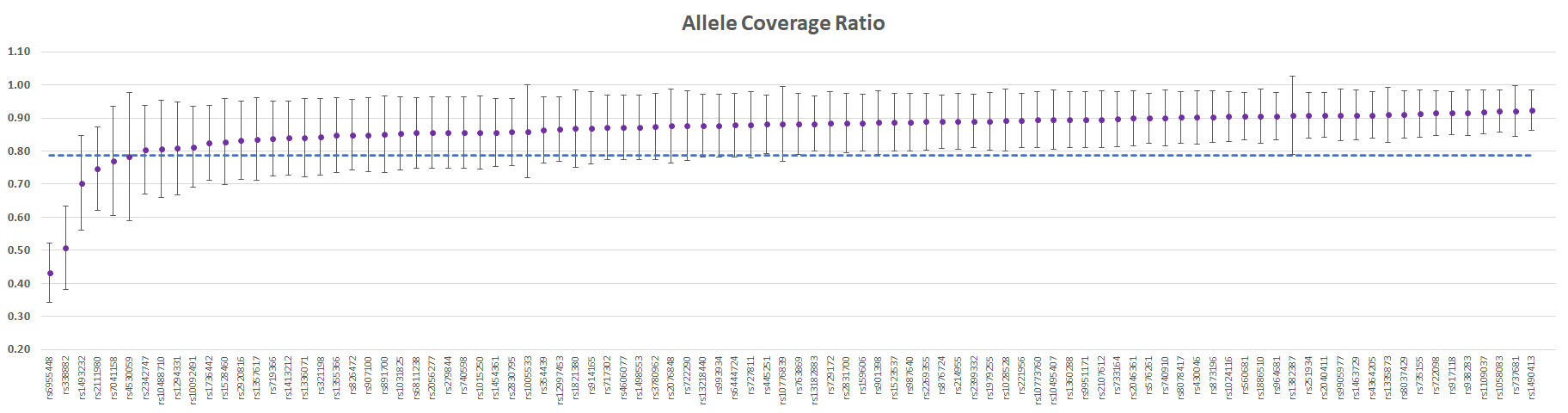

Supplement: Supplementary file 1 [file genes-14-01071-s001.zip › Figure S1 B.png]
